# Supplementary figures and images for: Anticonvulsant effects of novel and repurposed drugs on docetaxel-induced neuropathy in C. elegans
Source: PLoS One. 2026 Feb 6;21(2):e0342236. doi: 10.1371/journal.pone.0342236 (PMC12880658; doi:10.1371/journal.pone.0342236)

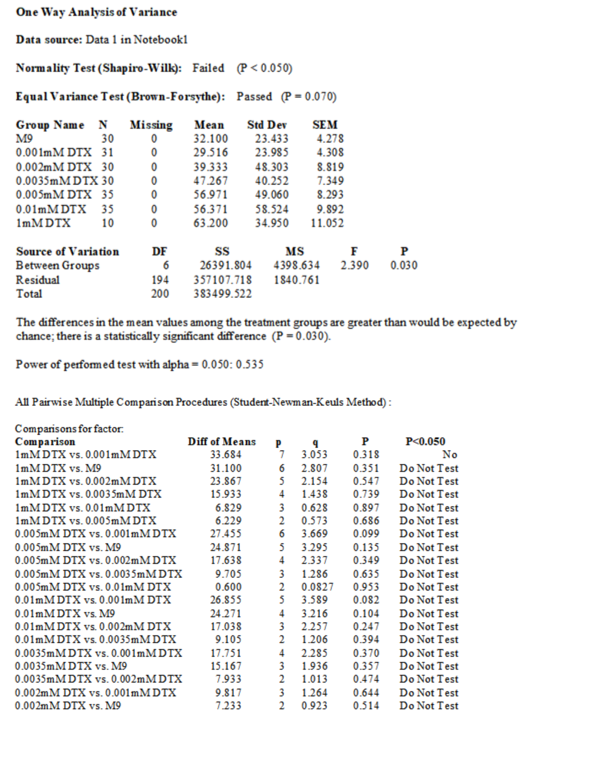

Supplement: S1 Fig — (TIF) [file pone.0342236.s001.tif]

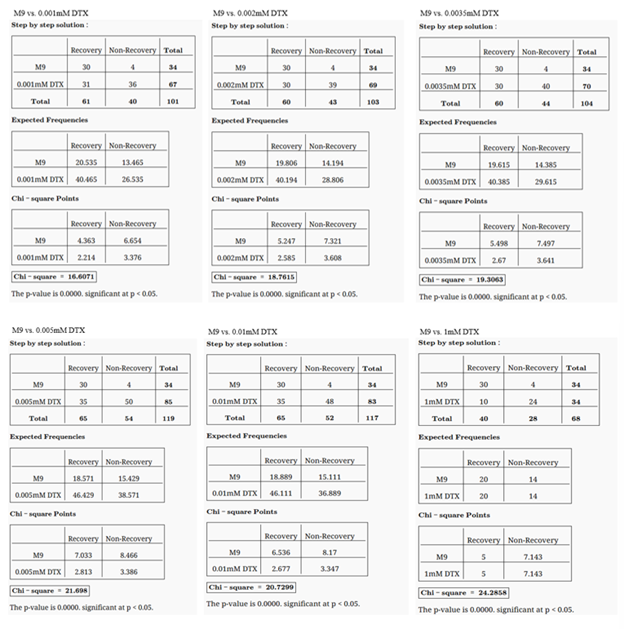

Supplement: S2 Fig — (TIF) [file pone.0342236.s002.tif]

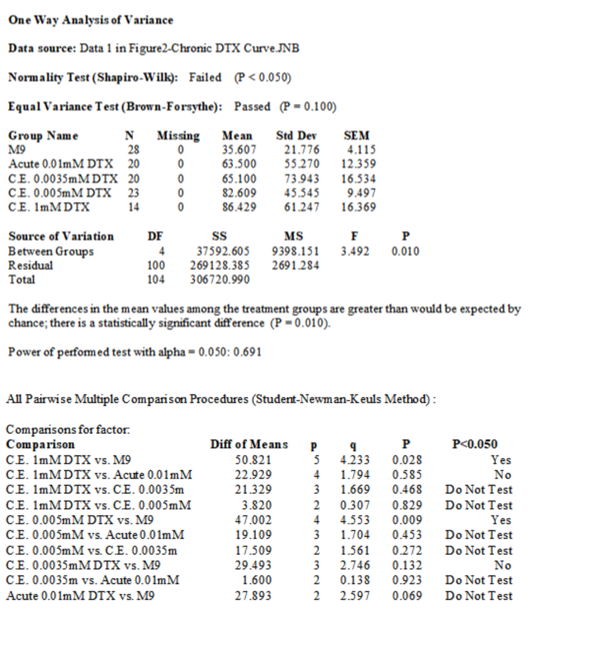

Supplement: S3 Fig — (TIF) [file pone.0342236.s003.tif]

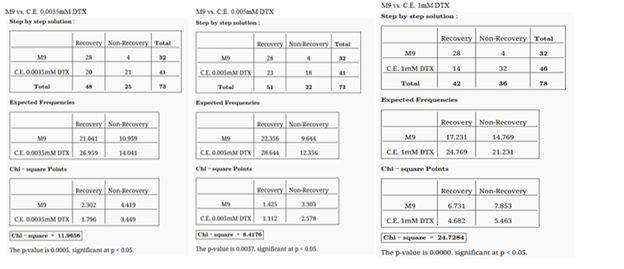

Supplement: S4 Fig — (TIF) [file pone.0342236.s004.tif]

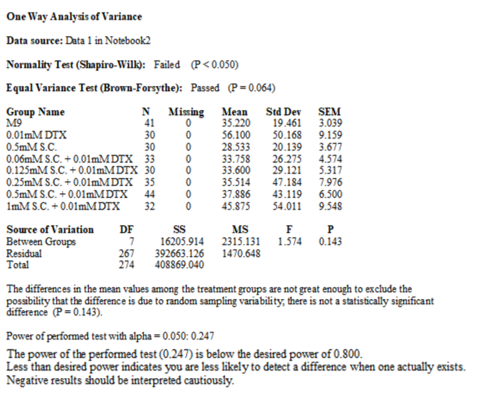

Supplement: S5 Fig — (TIF) [file pone.0342236.s005.tif]

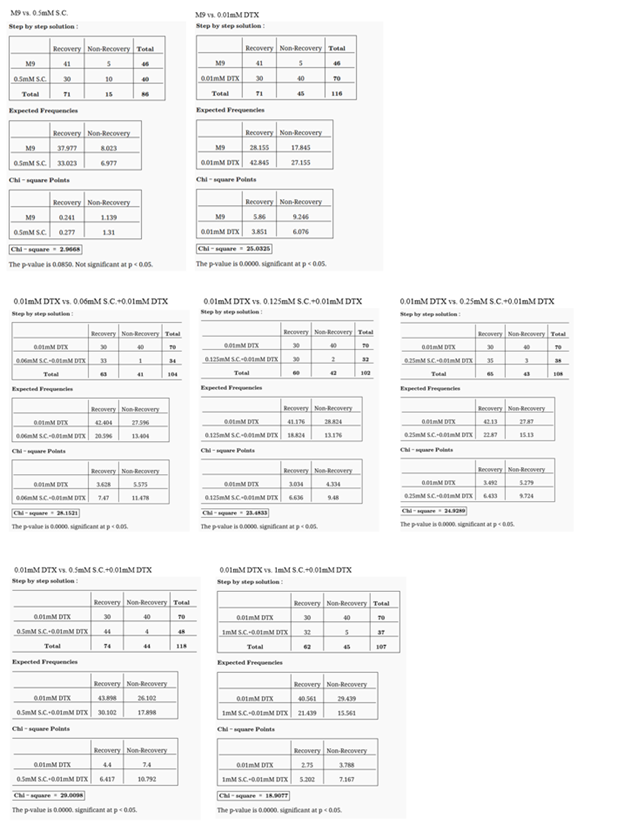

Supplement: S6 Fig — (TIF) [file pone.0342236.s006.tif]

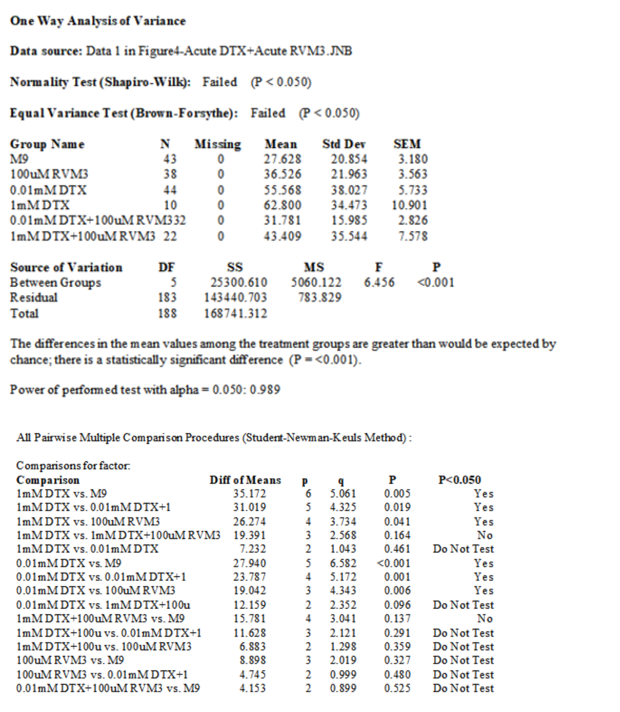

Supplement: S7 Fig — (TIF) [file pone.0342236.s007.tif]

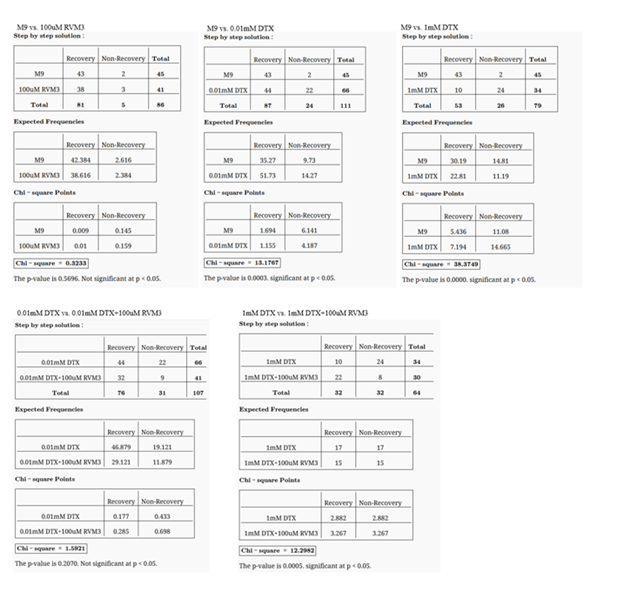

Supplement: S8 Fig — (TIF) [file pone.0342236.s008.tif]

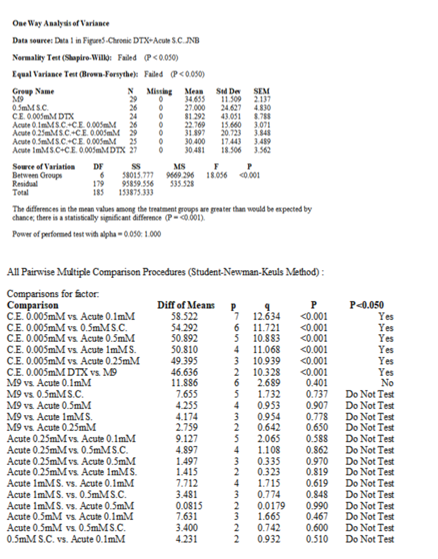

Supplement: S9 Fig — (TIF) [file pone.0342236.s009.tif]

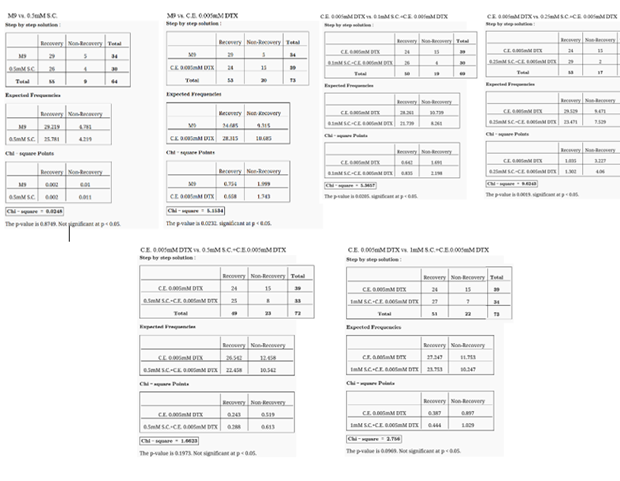

Supplement: S10 Fig — (TIF) [file pone.0342236.s010.tif]

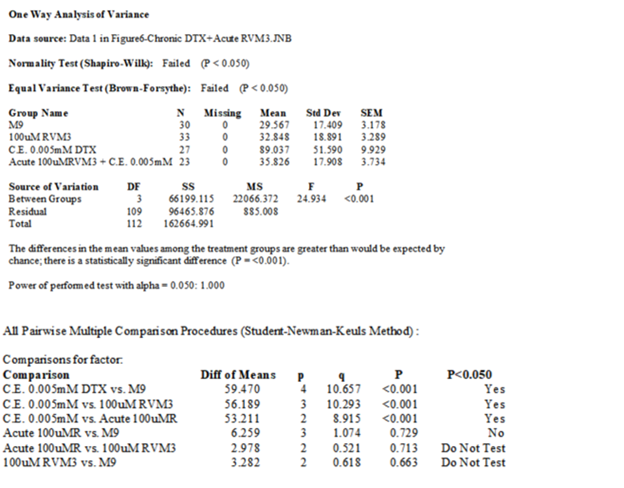

Supplement: S11 Fig — (TIF) [file pone.0342236.s011.tif]

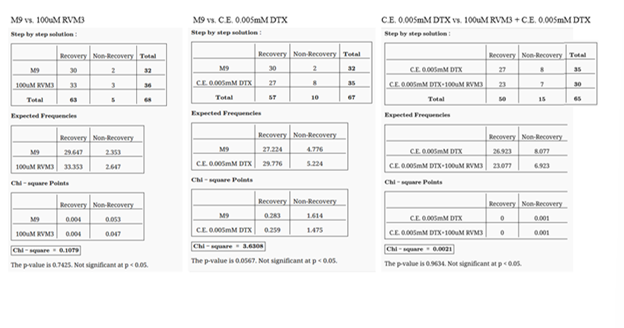

Supplement: S12 Fig — (TIF) [file pone.0342236.s012.tif]

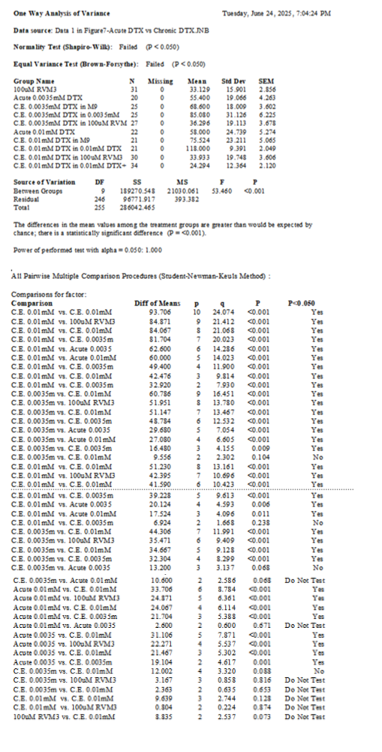

Supplement: S13 Fig — (TIF) [file pone.0342236.s013.tif]

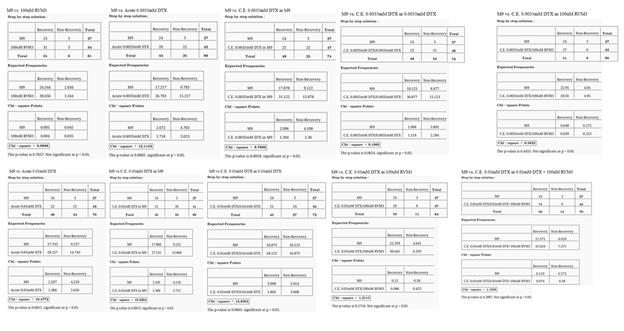

Supplement: S14 Fig — (TIF) [file pone.0342236.s014.tif]

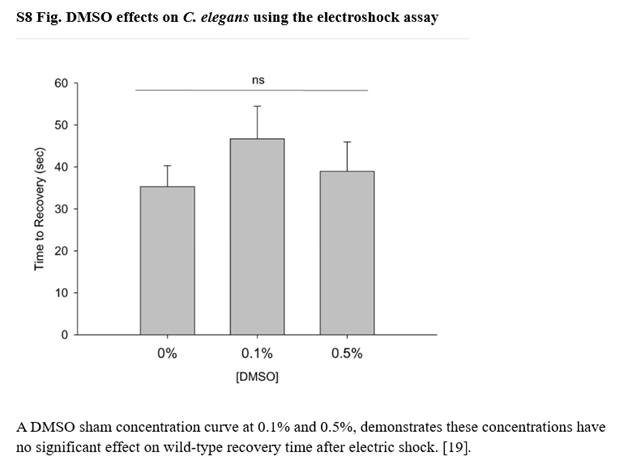

Supplement: S15 Fig — (TIF) [file pone.0342236.s015.tif]

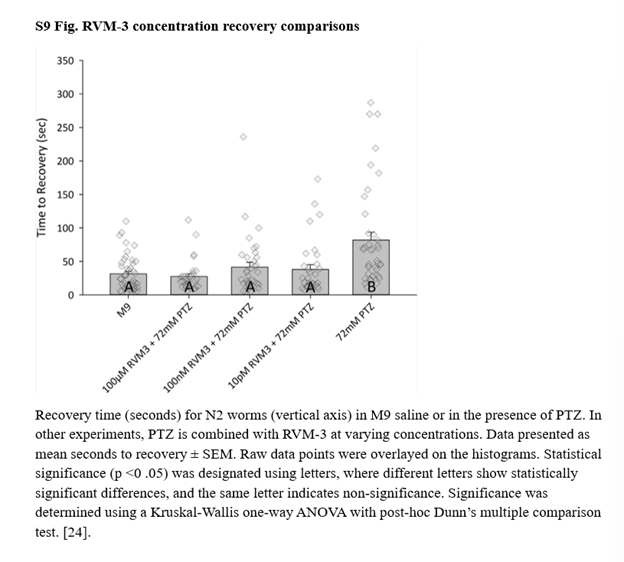

Supplement: S16 Fig — (TIF) [file pone.0342236.s016.tif]
