# Supplementary material for: Anticonvulsant effects of novel and repurposed drugs on docetaxel-induced neuropathy in C. elegans
Source: PLoS One. 2026 Feb 6;21(2):e0342236. doi: 10.1371/journal.pone.0342236 (PMC12880658; doi:10.1371/journal.pone.0342236)
Supplement: Attachment — Submitted filename: 4. ReviewerComments.docx [file pone.0342236.s017.docx]

Dear Reviewers,

We want to thank the reviewers for their thoughtful and constructive feedback. We have carefully considered each comment and revised the manuscript accordingly. We believe that the added clarifications and details have strengthened the manuscript. Below, we provide our detailed responses to each point.

**Reviewer 1:**

**Major Comments:**

**Acute exposure**

**1. What is the final concentration of DMSO for the docetaxel and sildenafil citrate that the worm experiences? It is not clear from the methods whether an equivalent volume of DMSO is used in the controls.**

In the acute condition exposures, we diluted docetaxel and sildenafil citrate in 1% dimethyl sulfoxide (DMSO) and subsequently diluted 1:10 in M9 saline. This methodology was also used for chronic condition exposures. This means that for acute condition exposures, the tested solutions contained M9 with 0.1% DMSO, meaning 0.1% of the total volume is DMSO and the remaining 99.9% is M9 saline. We note this detail under “chronic exposure protocol” **(Lines 107-111**). We also briefly reference this under the section titled “drugs and chemicals used” **(Lines 131-136)**, however we acknowledge we did not clarify this for the acute exposure protocol. We appreciate your attention to detail, as we have included this detailed description under the “acute exposure protocol” section **(Lines 102-105).**

**2. DMSO itself can have neuroprotective properties at low concentrations and needs to be included in your controls.**

Thank you for pointing this out. When we implemented DMSO as a dissolving vehicle for our compounds, we also considered the possibility that low concentrations of DMSO could generate neuroprotective effects, therefore, to address this issue, we turned to previously published data by our lab. In previous work, a concentration curve for dimethyl sulfoxide (DMSO) was conducted showing that there is no significant difference in recovery from electric shock in concentrations up to 0.5% DMSO [19]. We have included this dose curve image as a supplemental figure titled S8 Fig. DMSO effects on *C. elegans* using the electroshock assay. This is important since we accept that DMSO can be used as a solvent for this assay, without affecting the results observed by docetaxel and sildenafil citrate. For further clarification, we make a note of these results in the section titled “drugs and chemicals used” **(Lines 131-136).** We have also included the figure in this response for your accessibility. Considering that we had previously published work showing that a 0.1% concentration of DMSO did not show significant effects on the recovery-time of wild-type nematodes after electric shock, we did not run extra sets of controls in our work. If you feel like this is something that must be included in our current work, we would be happy to run the extra set of experiments. We like to note that we respectfully value your feedback on this matter and we seek to only justify our original methodology for this part of our study. However, if you do feel this is something that must be repeated we will address it without a problem.

A DMSO sham concentration curve at 0.1% and 0.5%, and demonstrates these concentrations have no significant effect on wild-type recovery time after electric shock [19]*.*

**Chronic exposure**

**3. With the chronic exposure experiments, however, you are dissolving solutions into your NGM plate. Therefore, the chronic drug concentration experienced by worm will by reduced by a factor dependent on the volume of the plate. If your plate has 10 mls of NGM in it, the stated concentrations of docetaxel will be reduced by a factor of 10. And that assumes that the plate volume is standardised (is it?)**

For chronic exposure experiments, we coated each plate with M9 saline containing the corresponding concentrations of docetaxel or vehicle control (M9 with 0.1% v/v dimethyl sulfoxide (DMSO). We mention this in the “chronic exposure protocol” section **(Lines 107-109).** In more detail, 10 milliliters of NGM agar were first dispensed per plate and once the agar solidified, 2 milliliters of corresponding solutions were poured on the surface of the agar plates. Plates were then covered and left to dry for 24 hours, away from light at room temperature, to avoid light and temperature degradation. All plates used in the chronic exposure protocol were standardized. After plates air-dried with the coated layer of corresponding solution, these were ready to use for experiments. New batches of plates were prepared every week to avoid drug degradation through time. For experiments, worms were placed on the surface of the plates. It is important to note that *C. elegans* do not burrow into the agar, but rather swim on the surface of the agar. A total of 6 gravid adult nematodes were transferred onto the surface of each coated plate and incubated for 2 days at 20 ℃. After 3 days of incubation, L4-stage worms were then transferred to a new drug-coated plate for overnight incubation at 22 ℃. On day 4, 1-day-old adult worms were incubated for 30 minutes in M9 saline (control) or the test solution prior to the electroshock delivery. We have included this detailed description of the protocol under the “chronic exposure protocol” section **(Lines 111-125).** Regarding acute docetaxel exposure, we observed that drug effects were concentration-dependent such that after reaching a threshold, the drug generated toxicity to the nematode which we observed as an increase in time-to-recovery from the shock-induced seizure-like behaviors **(Lines 323-326).** In the scenario of chronic drug exposure, we hypothesize that the effects observed were time-dependent with sensitization, not purely concentration effects. This due to the fact that chronic growth could have pre-disposed the nematode to be then affected by the subsequent acute drug exposure, resulting in toxicity seen as an increase in time-to-recovery from shock-induced seizure-like behaviors starting at lower concentrations **(Lines 326-329).**

**4. The authors need to clarify their experimental protocol and what the actual stated concentrations for the chronic experiments were**

We have included a detailed description of the chronic exposure protocol in the above response for Question 3. However, to further clarify, we have poured 2 milliliters of the prepared solutions onto the surface of each solidified agar plate. Plates were then covered and left to air-dry for 24 hours, away from light at room temperature to avoid light and temperature degradation. All plates used in the chronic exposure protocol were standardized. After plates air-dried with the coated layer of corresponding solution, these were ready to use for experiments. Additionally, for drug concentration specification, docetaxel was first dissolved in 1% DMSO, followed by a 1:10 dilution in M9 saline. Therefore, the tested solutions were dissolved in 0.1% DMSO of the total volume with a 99.9% M9 saline. This information was added under the “chronic exposure protocol” section **(Lines 107-111).**

**5. Wording in Figure 2 implies that chronic treatment “increases severity of behaviours”. But the statistics appear to be done only against chronic treatment of M9. Comparing Figures 1 and 2 – the data look pretty similar, indicating a lack of chronic response. Although given the point above about the chronic concentrations, this experiment may simply be replicating the acute effects. Do the authors have any evidence for an increase in behavioural severity?**

Figure 2 titled “Chronic docetaxel treatment increases severity of seizure-like behaviors”, is meant to show that nematodes exposed to a chronic docetaxel protocol, tend to display a concentration-dependent increase in time-to-recovery from a seizure-like behavior at lower concentrations (Figure 2), than those nematodes exposed to an acute docetaxel protocol (Figure 1). In figure 1, nematodes exposed to 0.0035 mM acute docetaxel experienced a time-to-recovery from seizure-like behaviors similar to worms exposed to M9 saline. However, nematodes exposed to chronic 0.0035 mM docetaxel experienced an increase in time-to-recovery from seizure-like behaviors that was significantly different from M9 saline. In our figures, we display statistical differences by using different letters. In figure 1, M9 saline and acute 0.0035 mM docetaxel were labeled “a”, indicating that both solutions do not display any significantly difference in time-to-recovery from seizure-like behaviors. In figure 2, M9 saline was labeled “a”, whereas chronic 0.0035 mM docetaxel was labeled “b” indicating a statistically significant difference in time-to-recovery from seizure-like behaviors between the two solutions.

To ensure clarity of the data displayed, we are changing the title for figure 2 to “Nematodes treated with chronic docetaxel, displayed an increase in time-to-recovery from seizure-like behaviors starting at lower concentrations” **(see Fig 2 in figure files and manuscript Lines 204-205).** In figure 2, we also compared the chronic exposure data to the acute exposure data. In this figure, we show that nematodes in M9 saline, column labeled “a”, displayed a statistically significant decreased time-to-recovery from seizure-like behaviors versus worms treated with chronic 0.0035 mM docetaxel labeled “b”. Furthermore, nematodes treated with chronic 0.005 mM and 1 mM docetaxel displayed a statistically significant increase in time-to-recovery different than animals exposed to chronic 0.0035 mM docetaxel and M9, therefore these columns were labeled “c”. As a form of comparison, we included a horizontal solid line in figure 2 to show that on average, nematodes exposed to acute 0.01 mM docetaxel displayed lower time-to-recovery from seizure-like behaviors than those animals exposed to chronic docetaxel concentrations. We indicate that different letters denote statistically significant difference in the mean values between the groups, and we also explain the meaning of each letter in each figure legend **(Lines 190-192, 207-210, 229-231, 251-252, 272-274, 288-289, 299-302).**

**6. Phrasing. The authors do not measure seizures or induction of seizures – they are specifically measuring time-to-recovery or lack of recovery – which is different. In a number of places the authors have misconstrued the experimental outcomes, which needs to be corrected. Specifically:**

We appreciate the reviewer for pointing this out. We agree that by mentioning the increase or decrease of seizure-like behaviors, we are conveying the incorrect idea. In this study, we are measuring time-to-recovery from a shock-induced seizure-like behavior, using our previously established electroshock assay [18-21]. Here, shock induces the seizure-like behavior, while exposure to M9 saline versus acute or chronic docetaxel affects the duration of time-to-recovery from the induced seizure-like behavior. Additionally, we are using sildenafil citrate and resveramorph-3 (RVM-3) to measure the effects these have in decreasing the duration of time-to-recovery from the shock-induced seizure-like behaviors once altered by exposure to docetaxel **(Lines 39-42)**. To clarify our methodology and results, we have addressed each of the scenarios pointed out by the reviewer below:

- **Line 140. A proconvulsant is an agent that initiates or lowers the threshold for seizures. The authors should either test for a proconvulsant effect (eg. Identify whether the voltage threshold for seizures is actually lowered) or reword for what is actually shown.**

In our “results section, our opening sentence states “We first tested whether docetaxel is a proconvulsant in *C. elegans.”* As the reviewer mentioned above, a proconvulsant is a substance that promotes convulsions by lowering seizure threshold in the brain, therefore increasing the risk for seizures. In this study, we did not use docetaxel as a proconvulsant, rather we employed the drug to measure its effects in modulating time-to-recovery from shock-induced seizure-like behaviors. We treated our nematodes in both acute and chronic conditions versus M9 saline to observe for the effects that docetaxel had on the time-to-recovery from the shock-induced seizure-like behaviors. To clarify our methodology and findings, we have reworded the phrasing to state “We first tested whether docetaxel is an agent that can increase time-to-recovery from shock-induced seizure-like behaviors in *C. elegans”* **(Lines 183-184).**

- **Line 176 states that “docetaxel models DIPN-seizure behaviour in the worm”. My understanding is that docetaxel is a proconvulsant and there isn’t evidence that it affects time-to-recovery from seizures. This statement needs to be supported or reworded.**

Docetaxel is not a proconvulsant but rather a taxane chemotherapeutic agent used to treat advanced, metastatic, or chemotherapy-resistant cancers, however its neurotoxic side effects often lead patients to abandon treatment [5, 6]. We mention this classification for docetaxel in our manuscript **(Lines 34, 55, & 58-61).** Docetaxel causes docetaxel-induced peripheral neuropathies (DIPNs) which includes motor neuropathy, tingling, muscle weakness, and numbness rather than acute or chronic seizures directly. This re-wording has been made in our manuscript **Lines 35-36.** Furthermore, in the past research has concluded that seizures following docetaxel use are rare and are not recognized as acute or chronic toxicity of the drug directly. However, a case study published in 1999 did report a patient developing an encephalopathy and subsequent seizures after receiving docetaxel [9]. This leads us to hypothesize that docetaxel may be used as an agent that may sensitize patients to develop seizures. In our study, we used the electroshock assay to model shock-induced DIPN-related muscle weakness *C. elegans*. We then treated nematodes with acute and chronic conditions of docetaxel to assess the effects of the drug in modulating time to recovery from shock-induced seizure-like behaviors. We have included this clarification in **lines 37-39**. Results obtained show that acutely or chronically exposing nematodes to docetaxel increases time to recovery from shock-induced seizure-like behaviors **(Lines 37-39)**. Specifically, nematodes treated with docetaxel exposure displayed an increase in time to recovery from shock-induced seizure-like behaviors [Fig1A]. We attribute these drug effects to be concentration dependent, such that after reaching threshold drug toxicity led to the display of increase in behavior duration (Lines 284-289). For chronic docetaxel exposure, we observed increase time to recovery from shcok-induced seizure-like behaviors starting at lower concentrations [Fig2A]. In this case, we attribute drug effects to be time dependent with sensitization, not purely concentration dependent effects **(Lines 328-329)**.

- **Line 197 specifically states “docetaxel induces seizure-like behaviors in C. elegans”, which is not supported by the results and needs to be reworded.**

We thank the reviewer for pointing this out. Docetaxel does not trigger seizure-like behaviors in our model. Instead it is the shock the one responsible for inducing the seizure-like behavior, while the docetaxel modulates the duration or time to recovery of the seizure-like behavior. We have re-worded this statement throughout the manuscript and we have also re-worded the sentence above **(Lines 201-203)**.

**Minor comments:**

**7. Methods line 94. The day after L4-stage worms would be Day 0 Adulthood (not Day 1).**

We revised our used protocol and we found that there was a typo on the days. On day 1, adult worms with eggs were plated on NGM agar plates seeded with OP50 *E. coli* for 2 days (rather than 3 days) at 20 ℃. On day 3, L4-stage worms were picked and plated on a new NGM agar plate seeded with OP50 *E. coli* for overnight incubation at 22 ℃. On day 4, 1-day-old adult worms were incubated for 30 minutes in M9 saline (control) or in the respective test solution prior to the electroshock delivery **(Lines 99)**. To stay consistent with our published protocol for the electroshock assay, we stay consistent with the numbering [20].

**8. The rationale for some experiments are not well explained. It isn’t clear to this reader why a neuroprotective effect of Viagra was expected. The authors should justify their choice of putative neuroprotective compounds.**

Being an FDA approved phosphodiesterase type 5 inhibitor, sildenafil citrate (SC), also known as Viagra works by selectively inhibiting the enzyme PDE5, triggering an increase in cyclic guanosine monophosphate (cGMP) levels, leading to smooth muscle relaxation and vasodilation. This information suggests that the agent may have neuroprotectant effects by improving blood flow and promoting neurogenesis. We also know that the NO-cGMP/PKG pathway is a potential target for seizure therapeutics, as it influences downstream K^+^ channel conductance through key players like PKG and PP2A [23]. SC works through the NO-cGMP/PKG pathway increasing cGMP levels, promoting vasodilation for neuronal conduction. Additionally, SC promotes K^+^ ion conductance through the same NO-cGMP/PKG pathway [23]. Furthermore, the effects of sildenafil have been linked to the GABAergic system [28], suggesting that sildenafil citrate reduces neuronal excitability through mechanisms involving both K^+^ channels and GABA signaling, ameliorating DIPN-related effects. For this reason we seek to re-purposed SC as a vasodilator that could be used as a neuroprotectant **(Lines 342-344)**.

**9. Figure legends state that “different letters denote a statistically significant difference in the mean values between the Groups” – but then it isn’t explained what “a” and “b” (and later on “a/b”) actually represent. I assume “a” is not significant and “b” is?**

In our manuscript we have included the following addition to our figure legends:

- Fig 1. Different letters denote a statistically significant difference in the mean values between the groups, where “a” stands for not statistically significantly different from M9 saline and “b” stands for statistically significantly different from M9 saline **(Lines 190-192).**
- Fig 2. Different letters denote a statistically significant difference in the mean values between the groups where “a” stands for not statistically significantly different from M9 saline, “b” stands for statistically significantly different from M9 saline, and “c” stands for statistically significantly different from M9 saline and solutions labeled “b” **(Lines 207-210).**
- Fig 3. Different letters denote a statistically significant difference in the mean values between the groups where “a” stands for not statistically significantly different from M9 saline, “b” stands for statistically significantly different from M9 saline, and “a/b” stands for not statistically significantly different from M9 saline or solutions labeled “b” **(Lines 229-231).**
- Fig 4. Different letters denote a statistically significant difference in the mean values between the groups where “a” stands for not statistically significantly different from M9 saline, and “b” stands for statistically significantly different from M9 saline **(Lines 251-252).**
- Fig 5. Different letters denote a statistically significant difference in the mean values between the groups where “a” stands for not statistically significantly different from M9 saline, and “b” stands for statistically significantly different from M9 saline **(Lines 272-274).**
- Fig 6. Different letters denote a statistically significant difference in the mean values between the groups where “a” stands for not statistically significantly different from M9 saline, and “b” stands for statistically significantly different from M9 saline **(Lines 288-289).**
- Fig 7. Different letters denote a statistically significant difference in the mean values between the groups where “a” stands for not statistically significantly different from M9 saline, “b” stands for statistically significantly different from M9 saline, “c” stands for statistically significantly different from M9 saline and other labeled solutions, and “d” stands for statistically significantly different form M9 saline and other labeled solutions **(Lines 299-302).**

**10. Figure 2. It is unclear why a line for 0.01 mM docetaxel is included when that isn’t being tested chronically.**

In figure 2, we compared the chronic exposure data to the acute exposure data. In this figure, we show that nematodes in M9 saline, column labeled “a”, displayed a statistically significant decreased time-to-recovery from shock-induced seizure-like behaviors versus worms treated with chronic 0.0035 mM docetaxel labeled “b”. Furthermore, nematodes treated with chronic 0.005 mM and 1 mM docetaxel displayed a statistically significant increase in time to recovery different than animals exposed to chronic 0.0035 mM docetaxel and M9, therefore these columns were labeled “c”. As a form of comparison, we included a horizontal solid line in figure 2 to show that on average, nematodes exposed to acute 0.01 mM docetaxel displayed lower time to recovery from seizure-like behaviors than those animals exposed to chronic docetaxel concentrations. The purpose of including the solid line for acute exposure was to show that chronic exposure introduced the time dependent effect with sensitization, not purely concentration dependent effects seen in acute exposure **(Lines 328-329)**.

**11. Figure 7. It isn’t completely clear here why 3.5 uM is being studied. Nor why it is listed as uM rather than mM (everywhere else in the manuscript).**

In acute exposure we tested 0.0035 mM docetaxel to show the upper limit of the drug before causing significantly different results when compared to M9 saline. In chronic exposure, we teste 0.0035 mM docetaxel to show the statistically significant difference that is generated following a continuous drug exposure. In figure 7, we wanted to compile all the results into a graph to show the effects of acute/chronic docetaxel versus the resveramorph-3 effects. In figure 7, we chose to use 3.5 uM rather than 0.0035 mM for visual clarity when generating the large graph.

**Reviewer 2:**

**1. The electroshock assay treats ~6 worms together per tube/shock. Report the number of independent tubes and independent experimental days per condition.**

We thank the reviewer for inquiring about this experimental detail, which we believe is very important for the validity of our study. For accurate screening, we ran six tubes per condition, each tube containing six nematodes. To avoid day effects on our screening, we ran two tubes per condition per day. For clarification, each day we included a trial of M9 saline as a negative control. We have included this detail in our manuscript **lines 168-171.**

**2. Acute test solutions contain 0.1% DMSO (drug dissolved in 1% DMSO then 1:10 in M9), but the stated acute control is M9 saline alone. Please include (and analyze against) an acute 0.1% DMSO vehicle control to match drug conditions; otherwise DMSO effects cannot be excluded.**

We appreciate your feedback. When we implemented DMSO as a dissolving vehicle for our compounds, we also considered the possibility that low concentrations of DMSO could generate neuroprotective effects, therefore, to address this issue, we turned to previously published data by our lab. In previous work, a concentration curve for dimethyl sulfoxide (DMSO) was conducted showing that there is no significant difference in recovery from electric shock in concentrations up to 0.5% DMSO [19]. We have included this dose curve image as a supplemental figure titled **S8 Fig. DMSO effects on *C. elegans* using the electroshock assay.** This is important since we accept that DMSO can be used as a solvent for this assay, without affecting the results observed by docetaxel and sildenafil citrate. For further clarification, we make a note of these results in the section titled “drugs and chemicals used” **(Lines 133-136).** We have also included the figure in this response for your accessibility. Considering that we had previously published work showing that a 0.1% concentration of DMSO did not show significant effects on the recovery-time of wild-type nematodes after electric shock, we did not run extra sets of controls in our work. However, if you feel like this is something that must be repeated and included in our current work, we would be happy to address it by running extra sets of DMSO experiments.

A DMSO sham concentration curve at 0.1% and 0.5%, and demonstrates these concentrations have no significant effect on wild-type recovery time after electric shock [19]*.*

**3. Docetaxel is hydrophobic; “coating NGM plates with M9 containing docetaxel” raises concerns about uniformity and stability. Please specify: volumes applied per plate, drying time, storage/light protection, time from coating to use, and evidence the compound distributes/retains bioactivity on agar. Similarly, RVM-3 is said to be dissolved directly in M9—provide rationale/solubility data, stock concentration, purity, and quality control. These details are critical for reproducibility.**

For chronic exposure, *E. coli*-seeded NGM agar plates were coated with M9 saline containing the specified concentration of docetaxel or vehicle control (M9 with 0.1% v/v DMSO; see next section). In detail, docetaxel was first dissolved in 1% DMSO, followed by a 1:10 dilution in M9 saline. Therefore, the tested solutions were dissolved in 0.1% DMSO of the total volume with a 99.9% M9 saline. As for plate preparation, 10-milliliters of NGM agar were first dispensed per plate and once the agar solidified, 2-milliliters of corresponding solutions were poured on the surface of the agar plates. Plates were then covered and left to dry for 24 hours, away from light at room temperature, to avoid light and temperature degradation. All plates used in this protocol were standardized. After plates air-dried with the coated layer of corresponding solution, these were ready to use for experiments. Ten plates were prepared per condition. New batches of plates were prepared every week to avoid drug degradation through time. Using the electroshock assay protocol, we tested each chronic plate per condition against our acute protocol to ensure compounds distributed evenly and retained bioactivity on the agar. This data was presented in each figure of the chronic protocols.

For experiments, nematodes were placed on the surface of each plate. It is important to note that *C. elegans* do not burrow into the agar, but rather swim on the surface of the agar. On day 1, six gravid adult worms were transferred onto the surface of each coated plate and incubated for 2 days at 20 ℃. On day 3, L4-stage worms were transferred to a new drug-treated plate for overnight incubation at 22 ℃. On day 4, 1-day-old adult worms were incubated for 30 minutes in M9 saline (control) or the test solution, prior to the electroshock delivery **(Lines 107-125).** In the case of RVM-3, this is a novel compound which has been synthesized and previously studied for solubility design by our team [22, 24]. RVM-3 has a molecular weight of 418.44 g/mol. For this study, lyophilized RVM-3 was stored in parafilm sealed amber glass vials away from light at 20°C for a period of up to three months. The day of experiments, RVM-3 was dissolved directly into M9 saline as performed and reported in our previous lab publications [24]. In detail, 1 milligram of the lyophilized compound was resuspended in M9 saline to make a stock concentration of 100 micromolar. RVM-3 solutions were stored in parafilm sealed amber glass vials away from light at 20°C for a period of up to two weeks. We used 100 micromolar concentration of RVM-3, since this concentration showed statistically significant effects in reducing time to recovery from shock-induced seizure-like behaviors in nematodes when compared to worms treated with 72mM pentylenetetrazol (pro-convulsant) alone [24]. We have included this dosage effect graph as a supplemental figure titled **S9 Fig. RVM-3 concentration recovery comparisons.** These details have been included in our manuscript **lines 136-142.**

Recovery time (seconds) for N2 worms (vertical axis) in M9 saline or in the presence of PTZ. In other experiments, PTZ is combined with RVM-3 at varying concentrations. Data presented as mean seconds to recovery ± SEM. Raw data points were overlayed on the histograms. Statistical significance (p <0 .05) was designated using letters, where different letters show statistically significant differences, and the same letter indicates non-significance. Significance was determined using a Kruskal-Wallis one-way ANOVA with post-hoc Dunn’s multiple comparison test. [24].

**4. The recovery criterion (three sinusoidal waves) is clear, but please state whether scorers were blinded to condition and how worms/tubes were randomized across treatments and days.**

To maintain drug screening and video analysis objectivity, two researchers were involved in collecting data for this study. Each researcher was responsible for completing randomly assigned dosage curves on different days of the week. For more details on experimental design, please refer to our added details to the manuscript in **lines 168-171.** To eliminate any bias during the video analysis process, videos were swapped between the two researchers and scores were blinded to condition **(Lines 171-175).**

**5. You repeatedly link seizures to Docetaxel-Induced Peripheral Neuropathies (DIPNs). Seizures are central phenomena; while docetaxel-associated encephalopathy and seizures are reported, seizures are not typically considered a feature of peripheral neuropathy per se. Please reframe as docetaxel-associated neurotoxicity (which can include seizures) rather than “DIPN-related seizures,” unless you provide primary evidence/mechanism tying the peripheral pathology to seizure generation.**

We really appreciate the reviewer for mentioning this. We acknowledge that by calling the observed behaviors a docetaxel-induced peripheral neuropathy we are linking this effect to seizures produced by docetaxel, which is an incorrect idea. We know that a proconvulsant is a substance that promotes convulsions by lowering seizure threshold in the brain, therefore increasing the risk for seizures. Docetaxel causes docetaxel-induced peripheral neuropathies (DIPNs) which includes motor neuropathy, tingling, muscle weakness, and numbness rather than acute or chronic seizures directly. This re-wording has been made in our manuscript **Lines 35-36.** Furthermore, in the past research has concluded that seizures following docetaxel use are rare and are not recognized as acute or chronic toxicity of the drug directly. However, a case study published in 1999 did report a patient developing an encephalopathy and subsequent seizures after receiving docetaxel [9]. This leads us to hypothesize that docetaxel may be used as an agent that may sensitize patients to develop seizures. In this study, we did not use docetaxel as a proconvulsant, rather we employed the drug to measure its effects in modulating time-to-recovery from shock-induced seizure-like behaviors. We treated our nematodes in both acute and chronic conditions versus M9 saline to observe for the effects that docetaxel had on the time-to-recovery from the shock-induced seizure-like behaviors. To clarify our methodology and findings, we have reworded the phrasing to state “We first tested whether docetaxel is an agent that can increase time-to-recovery from shock-induced seizure-like behaviors in *C. elegans”* **(Lines 183-184).**

**6. The Results text states that RVM-3 significantly reduced %NR with both 0.01 mM and 1 mM acute docetaxel, but the figure legend/statistics indicate significance only at 1 mM (0.01 mM + RVM-3 p≈0.21, n.s.).**

This correction has been made on the manuscript **(Lines 253-254)** and on the figures file under Fig4 legend.

**7. You describe concentration dependence qualitatively. Please include dose–response curves with fitted models where possible (even exploratory), report EC50/IC50 or at least slope parameters, and show per-tube datapoints.**

We thank the reviewer for recommending inclusion of dose–response curves and associated model fitting. We agree that such analyses would provide additional quantitative insight. However, the primary aim of this study is to establish and demonstrate the utility of our electroshock assay as a screening platform rather than to generate complete pharmacological profiles for each compound. Producing full dose–response datasets and fitting EC50/IC50 models would require additional analyses that fall outside the scope of the present work. To address the reviewer’s point, we have revised the manuscript to clearly state that our concentration-dependent assessments are qualitative and exploratory, and we now acknowledge the need for future studies to perform a full dose–response characterization **(Lines 385-391).** We hope these clarifications are acceptable to the reviewer.

**Minor issues**

**8. Mechanistic claims: Tone down language implying mechanism (e.g., “RVM-3 potentially acts as an irreversible agonist by covalently binding…”) unless direct evidence is presented here.**

Likewise, sildenafil’s effects on K⁺ channels are likely indirect via cGMP/PKG; keep causal language cautious and clearly attributed to prior work.

We have addressed these comments in our manuscript as indicated by the reviewer **(Lines 241-246, 337-339).**

**9. Terminology consistency: Use “seizure-like behavior” throughout for C. elegans; avoid unqualified “seizures”.**

We have addressed our terminology throughout the manuscript to keep it consistent and accurate by mentioning “seizure-like behaviors” rather than seizures. We have also reworded to clarify that these are shock-induced seizure-like behaviors, where docetaxel is an agent screened for the effects in modulating time to recovery from these shock-induced seizure-like behaviors.

**10. Ensure DTX not “DTC” (typo appears once in a legend).**

We have corrected this typo in **line 237** of our manuscript as well as in the figure 3 legend in our figures document.

**11. Standardize µM/mM spacing.**

We have revised this in our figure legends.

**12. Citations: Consider citing C. elegans cGMP/PKG genetics (e.g., egl-4; endogenous pde genes), to support the proposed pathway testing in future work.**

We have included these citation in our manuscript, **line 383.**

**Reviewer 3:**

**Minor suggestions:**

**1. Without having to read papers cited( 18-21 ) can the authors give a little bit of background in this manuscript why electroshock is a good model to induce seizures.**

*C. elegans* possesses 302 neurons, conserved neurotransmitter systems, a simplified neuronal circuitry, and a fully mapped connectome, making it a powerful model for neurological studies. Building on our previous work [20], we established an electroshock assay for pharmacological screening. This assay elicits a shock-induced seizure-like behavior that serves as a behavioral indicator of locomotor circuitry and neuronal function, enabling the identification of compounds that influence recovery time from these behaviors **(Lines 144-149).**

**2. I am not sure of the logic to try Sildenafil citrate as compared to more specific seizure reducing medications like levetiracetam or the various other medications. The authors state Sildenafil citrate works in the C. elegans model to reduce the seizure induced by docetaxel. But does Sildenafil citrate reduce the induction of the electroshock itself ? So then the seizure is already dampened to reduce the effect of the electroshock but not necessarily the effect of docetaxel ?**

We thank the reviewer for this question. In this study, we measured time-to-recovery from a shock-induced seizure-like behavior using our previously established electroshock assay [18–21]. In this assay, the shock elicits the seizure-like behavior, and exposure to M9 saline or to acute or chronic docetaxel alters the duration of recovery. We also assessed the effects of sildenafil citrate and resveramorph-3 (RVM-3) on recovery time after docetaxel exposure **(Lines 39–42).** As shown previously [20], a 47 V shock produces a recovery time at approximately 50% of the maximal response. In the current study, acute and chronic docetaxel exposure increased recovery time **(Lines 321–329)**. Sildenafil citrate significantly reduced recovery time following shock-induced seizure-like behaviors [Fig. 3, 5], consistent with our prior findings that sildenafil citrate decreases recovery time in this assay [29]. Based on these observations, we interpret sildenafil citrate as a compound that reduces recovery time in this behavioral paradigm without evidence of altering shock induction **(Lines 344-346).**

**3. In the Discussion it is stated that Docetaxel-Induced Peripheral Neuropathies (DIPNs) while will also cause seizures- related to humans.
So, it is not known if Docetaxel induces seizures in C. elegans or did I miss something ? Or are the seizures only induced by electroshock in this model with ones exposed to Docetaxel?**

In this study, we are measuring time-to-recovery from a shock-induced seizure-like behavior, using our previously established electroshock assay [18-21]. Here, shock induces the seizure-like behavior, while exposure to M9 saline versus acute or chronic docetaxel affects the duration of time-to-recovery from the induced seizure-like behavior. Also, sildenafil citrate and resveramorph-3 (RVM-3) are used to measure the effects these agents have in decreasing the duration of time to recovery from the shock-induced seizure-like behaviors once altered by exposure to docetaxel **(Lines 39-42)**. Furthermore, we acknowledge that docetaxel is not a pro-convulsant therefore, it does not directly induce seizures in humans nor nematodes. Docetaxel causes docetaxel-induced peripheral neuropathies (DIPNs) which includes motor neuropathy, tingling, muscle weakness, and numbness rather than acute or chronic seizures directly. This re-wording has been made in our manuscript **Lines 35-36.** In this study, we did not use docetaxel as a proconvulsant, rather we employed the drug to measure its effects in modulating time-to-recovery from shock-induced seizure-like behaviors. We treated our nematodes in both acute and chronic conditions versus M9 saline to observe for the effects that docetaxel had on the time-to-recovery from the shock-induced seizure-like behaviors. To clarify our methodology and findings, we have reworded the phrasing to state this throughout our manuscript.

**4. It could strengthen the article by including some discussion about the potential side effects or toxicity of sildenafil citrate and Resveramorph-3 in C. elegans, since understanding any harmful or side effects is important when considering these compounds as treatments.**

In terms of toxicity, from our findings [Fig 3, 5] we observed that acute co-exposure to high-dose sildenafil citrate and 0.01 mM docetaxel increased seizure-like behaviors, whereas chronic docetaxel exposure followed by acute sildenafil treatment did not produce this effect. This pattern suggests a time-dependent interaction in which simultaneous exposure may transiently heighten sensitivity to seizure-like behaviors, while chronic docetaxel exposure may induce adaptive responses that mitigate this sensitivity. The underlying mechanism remains unclear and warrants further investigation **(Lines 348- 355).** In the case of RVM-3, acute exposure to 100 µM RVM-3 in combination with docetaxel decreased the duration of shock-induced seizure-like behaviors [Fig 4]. Nematodes grown chronically in docetaxel and then treated acutely with RVM-3 showed a similar reduction [Fig 6]. These findings suggest that RVM-3 can reduce recovery time from seizure-like behaviors under both acute and chronic conditions. One interpretation is that the pathways or processes influenced by RVM-3 remain responsive regardless of prior docetaxel exposure; however, the basis of these effects is not yet understood. RVM-3 is a novel compound and therefore further studies are required to evaluate its potential toxicity, define dose-dependent effects, and determine whether the observed reductions in recovery time reflect specific actions within this behavioral paradigm or broader effects on neuronal or locomotor function **(Lines 361-370).**
