# Supplementary material for: Anticonvulsant effects of novel and repurposed drugs on docetaxel-induced neuropathy in C. elegans
Source: PLoS One. 2026 Feb 6;21(2):e0342236. doi: 10.1371/journal.pone.0342236 (PMC12880658; doi:10.1371/journal.pone.0342236)
Supplement: Attachment — Submitted filename: Response to Reviewers.docx [file pone.0342236.s018.docx]

Dear Reviewers,

We want to thank the reviewers for their continuous support and constructive feedback. We have thoroughly considered each comment and revised the manuscript accordingly. We believe that the added clarifications and details have continued to strengthen the manuscript. Below, we provide our detailed responses to each point.

**Reviewer 1:**

**Major Comments:**

**Acute exposure**

1. **That’s fine.**
2. **Essentially that is fine – and I am certainly not going to require you to redo your controls in light of the provided data. But I would suggest in future always doing your controls in the equivalent volume of solvent. I note in the figure provided that the mean is increasing in 0.1% DMSO (from ~35 to 45 sec) with fairly large error bars. And it may well be that the solvent is a contributing factor to the statistical effect seen in this manuscript.**

Thank you for your feedback on this matter and we appreciate that you accept the supporting data we have presented. After revisiting our data and the data previously collected in our lab, we understand the importance of reporting controls that are equivalent to the volume of the solvent used. This methodology indeed clarifies any discrepancies that may arise in future reported data. For future investigations, we will ensure to include controls accordingly.

**Chronic exposure**

3. **&	4. I am still unclear on the final concentrations for the chronic exposure experiments and think this needs to be clarified explicitly in the text. From the revised wording, the drugs are dissolved in 1% DMSO and then diluted 1/10 in M9 (equivalent to the acute experiments). For the chronic experiments, this is further dissolved in 10 mls NGM (+ 2 mls solution = 12 mls total). The wording certainly implies that the final DMSO concentrations in the chronic experiments will not be 0.1% - they will be ~0.01%. And it should also be clarified that the final drug concentrations are taking the additional volumes into account as well.**

Thank you for pointing this out. After studying our calculations, we have revised our manuscript to include these specifications in lines 110, and 114-117. We acknowledge that the different DMSO concentrations used for acute versus chronic experiments do not generate a direct comparison between both models.

**5. This is worded a little better. However, the authors are still implying that “chronic treatment” is different from “acute treatment” by using the phrase “starting at lower concentrations – without overtly saying lower to what? Clearly that is meant to imply lower concentrations to the acute experiments in Figure 1. But there are no statistical comparisons between acute and chronic treatments – indeed the comparisons could not be direct. Your ANOVA in Figure 1 is using more conditions than the ANOVA in Figure 2 and may be affecting the significance. Additionally (see points 3-4 above), I am still unclear that the DMSO concentrations are equivalent for both acute and chronic treatments. I remain suspicious that there is no statistical (let alone biological) effect of chronic treatment in comparison to acute treatment.**

In our manuscript, we clarify that differences in plate preparation resulted in different DMSO concentrations between chronic and acute exposure experiments (Lines 110, 114-117), making these two methods not directly comparable, as no direct statistical comparison was performed. Additionally, we have changed the description title for Fig 2 to indicate that “Chronic exposure to docetaxel increased time to recovery from shock-induced seizure-like behaviors [Fig 2A] and % NR [Fig 2B], in contrast to nematodes exposed to M9 saline, which did not exhibit such changes” (Lines 205-210 & 332-333). In our Supplemental Figures document titled “S1 Appendix” we have also included this correction (Lines 403-408).

**6 - 9. That’s fine.**

**10. The line for acute 0.01 mM docetaxel in Figure 2 should be removed as it is meaningless in the context of the chronic experiment. And indeed the acute 0.01 mM docetaxel from Figure 1A is clearly under 60sec whereas the line in Figure 2A is over 60 sec – so I don’t know where this number is actually generated from?**

We have removed the line for acute 0.01 mM docetaxel in Figure 2. This change can be observed in the Figures document submitted with this revision.

**11. That’s fine.**

**Reviewer #2: The authors have substantially improved the methods transparency e.g. the number of tubes/condition and the blinding procedures, while also correcting results/legend inconsistency.**
